# Relationship between addictions and obesity, physical activity and vascular aging in young adults (EVA-Adic study): a research protocol of a cross-sectional study

**DOI:** 10.3389/fpubh.2024.1322437

**Published:** 2024-01-26

**Authors:** Sara Vicente-Gabriel, Cristina Lugones-Sánchez, Olaya Tamayo-Morales, Alberto Vicente Prieto, Susana González-Sánchez, Sandra Conde Martín, Marta Gómez-Sánchez, Emiliano Rodríguez-Sánchez, Luis García-Ortiz, Leticia Gómez-Sánchez, Manuel A. Gómez-Marcos, EVA-Adic Investigators Group

**Affiliations:** ^1^Primary Care Research Unit of Salamanca (APISAL), Salamanca Primary Care Management, Institute of Biomedical Research of Salamanca (IBSAL), Salamanca, Spain; ^2^Intensive Care Unit, Salamanca University Hospital, Salamanca, Spain; ^3^Research Network on Chronicity, Primary Care and Health Promotion (RICAPPS), Barcelona, Spain; ^4^Salamanca Primary Care Management, Castilla and León Health Service–SACYL, Salamanca, Spain; ^5^Home Hospitalization Unit, Marqués de Valdecilla University Hospital, Santander, Spain; ^6^Department of Medicine, University of Salamanca, Salamanca, Spain; ^7^Department of Biomedical and Diagnostic Sciences, University of Salamanca, Salamanca, Spain; ^8^Emergency Department, La Paz University Hospital, Madrid, Spain

**Keywords:** substance-related disorders, gambling, internet use, technology addiction, obesity, healthy lifestyle, physical activity, vascular stiffness

## Abstract

**Background:**

Behavioral and substance addictions are prevalent health problems that, alongside obesity, are linked to reduced physical activity and increased sedentary time. Similarly, arterial stiffness and vascular aging are processes that begin gradually at an early age and are closely associated with morbidity and mortality from cardiovascular diseases. The main objective of this study is to analyze how addictions are related to obesity and body fat distribution, physical activity, sedentary time, arterial stiffness and vascular aging, as well as sleep quality, cognitive function and gender differences in young adults aged between 18 and 34 years.

**Methods:**

This cross-sectional descriptive observational study will analyze data from 500 subjects (250 men and 250 women) aged 18–34 without cardiovascular disease, selected by simple random sampling with replacement from the urban population of the city center of Salamanca (34,044 people aged 18–34, with 18,450 women and 15,594 men). Behavioral and substance addictions, as well as sleep quality and cognitive impairment will be assessed using questionnaires. The Pittisburg Sleep Quality Index (PSQI) will be used to measure sleep quality and the Ford questionnaire will be used to measure insomnia in response to stress. For obesity, weight, height, waist and hip circumference, body composition will be measured with the *Inbody 230*^®^ impedance meter. For physical activity and sedentary time, we will use the *Actigraph*^®^ accelerometer alongside the international physical activity questionnaire (IPAQ) and the Marshall questionnaire. The *Sphygmocor System*^®^ will be used for pulse wave analysis and carotid-femoral pulse wave velocity (cfPWV), while the *Vasera VS-2000*^®^ will measure cardio ankle vascular index (CAVI) and brachial-ankle pulse wave velocity (baPWV). Vascular aging will be calculated with the 10th and 90th percentiles of cfPWV or baPWV. Demographic, analytical variables will be collected, as will data to assess vascular, cardiac, renal, and brain injury.

**Discussion:**

Addictions are on the rise in today’s society, affecting the mental health and well-being of those who suffer from them, generating important social problems such as job loss, family dysfunction, debt and social isolation. Together with obesity, they are prevalent health problems in young adults and are associated with lower physical activity and higher sedentary time. Meanwhile, arterial stiffness and vascular aging are processes that begin gradually at an early age and determine morbidity and mortality caused by cardiovascular diseases. The results of this project will allow us to understand the situation regarding behavioral and substance addictions in young adults. Better understanding of these addictions will in turn facilitate the development of more effective prevention strategies and intervention programs, which can then reduce the negative impact at both the individual and societal levels.

**Clinical trial registration:**

[ClinicalTrials.gov], identifier [NCT05819840].

## 1 Introduction

Behavioral addictions cause loss of control and inability to stop or control addictive behavior, and despite their negative impacts on mental health, they are increasingly prevalent. For these reasons, the National Strategy on Addictions 2017–2024 has incorporated non-substance or behavioral addictions as a new field, with special emphasis on gambling (in-person or online) and addictions through new technologies (1). Thus, gambling with money and the use of Internet, smartphones and video games are common activities among young adults, due partly to the immediacy of reward, ease of access and anonymity they offer (1, 2). These disorders usually begin in adolescence, affecting behavior patterns and causing significant deterioration in personal aspects (1, 3). In Spain, behavioral addictions have increased during the last decade. It is estimated that 58% of the population aged between 15 and 64 years has participated in gambling for money in the last year, with in-person gambling more frequent than online gambling, while 97% of the population have used the internet for recreational purposes during the last month (1). Compulsive internet use has also increased (from 2.9% in 2017 to 3.5% in 2022) and 50% of young people play video games at least weekly (1). These behaviors vary by sex: gambling with money is more frequent among men, while compulsive Internet use is higher in women (1). These behaviors vary by sex: gambling with money is more frequent among men, while compulsive Internet use is higher in women (4, 5). Due to lockdown and social restrictions, these types of behaviors increased during the confinement as a coping strategy for stress or anxiety (6, 7).

Legal substances continue to be the most used substances. According to the latest data published in the EDADES survey, in 1995–2022, 93% of the Spanish population between 15 and 64 years of age reported drinking alcohol and 70% said they had smoked tobacco at some point in their lives, with consumption starting around the age of 16 and being higher in men (8, 9). In addition, drinking alcohol can act as a gateway to other substances (1). In Spain, 33.1% of the population aged between 15 and 64 smoke daily (10). In addition, the use of other modalities such as vaping has increased, especially among 15- to 24-year olds (2). The use of psychoactive substances is higher in men, except for hypnosedatives, which is higher in women (8, 11). In Spain, the use of illegal substances (cannabis, the most widespread, cocaine and ecstasy) has increased in recent years, with a prevalence of 40% for cannabis and 5.1% for ecstasy, at 18 years of age (8, 11, 12). Polydrug use compounds the risks, increasing mental illnesses (10). A recent study found that gender, age, and dual pathology had an impact on substance use and mental health during the pandemic by COVID-19. Men had greater alcohol and cocaine use, while women experienced more depressive and anxiety symptoms. In addition, younger adults were using cocaine and cannabis more frequently, and had more socio-familial and legal problems. Finally, dual pathology was related to the use of benzodiazepines, work problems (unemployment) and anxiety-depressive symptoms. The differences found (in age, gender and dual pathology) should be taken into account when planning health measures in this population (13).

Both general and abdominal obesity are related to an increase in deaths from all causes, being one of the main cardiovascular risk factors (14, 15). Obesity has been on the increase ever since records began (16). According to the WHO, it has almost tripled since 1975; in 2016, more than 1.9 billion adults worldwide aged over 18 were overweight and more than 650 million adults were obese (16). This increase also affects children and adolescents, so between the ages of 5 and 19 it has increased drastically from only 4% in 1975 to more than 18% in 2016 (16). In Spain, this prevalence is 21.6% (22.8% in men and 20.5% in women), with the prevalence of abdominal obesity being 33.4% (23.3% in men and 43.4% in women) (17). This has raised mortality by more than 50%, making it the fourth most preventable factor reducing quality of life (18). This increase is related from early ages to less physical activity, and more sedentary time in front of screens (19, 20).

Physical activity improves quality of life, which benefits the health system and society (21, 22). Thus, most international (23, 24), recommending at least 150 min/week of moderate-high intensity physical activity to obtain benefits. However, physical activity has decreased and sedentary lifestyle and daily time in front of screens have increased (24, 25), raising cardiovascular risk and general mortality (26, 27). Physical activity at younger ages can have positive health effects in adulthood (28, 29). Despite these health benefits, young adults and adolescents sit for many hours a day and many are physically inactive (30). Furthermore, the use of smartphones and other technologies is more prevalent in these age groups (31, 32), assuming an increase sedentary lifestyle (33).

Increased arterial stiffness occurs with a reduction in the elasticity of the arteries, and is a good risk predictor for cardiovascular diseases (CVD) just as important that traditional cardiovascular risk factors (34). Thus, arterial stiffness measured non-invasively has been positively associated with cardiovascular events (35, 36). It is linked to the appearance of vascular aging (37, 38), reflecting the dissociation between the chronological and biological age of the large arteries, with such abnormalities preceding the appearance of cardiovascular events (37, 39). In recent decades, epidemiological studies have been carried out to clarify the determining factors of vascular aging (40), and its study has aroused great interest (37, 38). Vascular aging is a gradual process that begins at an early age and reflect biological aging (37, 41, 42). The advantages of physical activity are known (38). However, the influence of addictions during youth on vascular aging has been little studied. The study of vascular aging in this life stage has aroused great interest in recent decades because it shows a better relationship with morbidity and mortality from cardiovascular diseases than biological aging (37, 38).

These changes in habits also affect other aspects, such as sleep, the duration of which is related with cardiovascular problems (43). Currently, insomnia or unsatisfactory sleep has a prevalence of approximately 6–10% in industrialized countries, with women more affected than men (44). The first meta-analysis to report on the prevalence of mental problems in Spain during the COVID-19 crisis showed that the prevalence of insomnia was 57%, with the majority being young adults (45), while the average European rate was lower (30.8% % IC, 27.1–34.4%) (46). This high prevalence of sleep disorders may be because they constitute a vulnerable group in continuous change due to maturation, academic and work processes (47). It is also a population that makes greater use of smartphones, the Internet, social networks and video games and presents greater substance use, factors that are closely linked to insomnia and anxiety (43, 48). The combination of sleep disorders with these types of addiction thus affects their quality of life (49).

For these reasons, the main objective of this study is to analyze the links between addictions and obesity, the distribution of body fat, physical activity, sedentary time, arterial stiffness and vascular aging, alongside sleep quality, cognitive function and gender differences in young adults.

The secondary objective is to analyze how the quality and quantity of sleep is associated with the appearance of addictions, obesity, level of physical activity, sedentary time, cognitive function and arterial stiffness, as well as differences by gender.

## 2 Methods and analysis

### 2.1 Study design and context

This is a descriptive observational study of cases and controls in which the differences between subjects with (cases) and without (controls) behavioral and/or drug addictions with obesity, physical activity, sedentary lifestyle, arterial stiffness and vascular aging, and the relationships between them, will be analyzed. The study will be carried out in the urban center of Salamanca, in the Primary Care Research Unit (APISAL), involving researchers from three groups of the IBSAL.

This project was approved by the Committee of ethics of research with medicines of the health area of Salamanca on 7/10/2021 (CEIm Reference Code Ref. PI 2021 088671048), and on 07/24/2023 (Reference Code CEIm reference Ref. PI 2023 071332). The SPIRIT (Standard Protocol Items: Recommendations for Interventional Trials) checklist 1/4/2024 11:04:00 a.m. is available for this protocol (Supplementary material 1).

### 2.2 Study population

An urban population from the health area of Salamanca will be recruited by means of stratified random sampling by age and sex groups with replacement, based on the individual health card (TIS), 500 subjects aged between 18 and 34 years will be selected.

Candidates will be invited to participated by phone, if they accept, the study visit will be scheduled according to their availability. They will be considered non-responsive if three calls are made in three different days at different times of the day (morning, noon and afternoon) and they do not answer.

Inclusion criteria: Patients between 18 and 34 years of age who agree to participate in the study and do not meet any exclusion criteria. Exclusion criteria: terminally ill subjects who are unable to travel to the health centers to undergo the corresponding examinations and who do not sign the consent form. The selected questionnaires will be used to determine whether the participant has any type of addiction.

**Figure 1:**
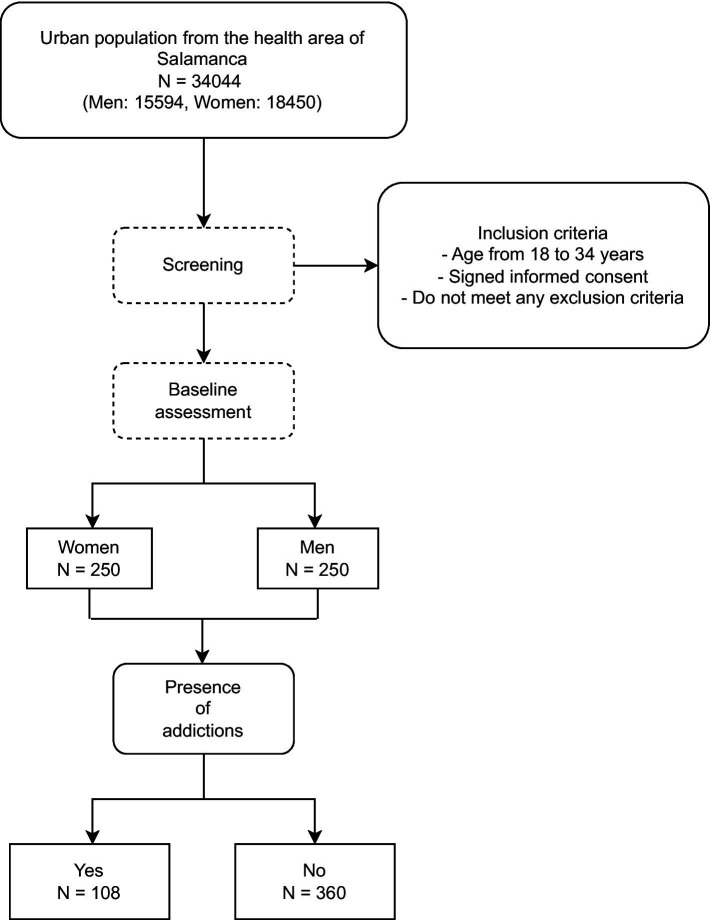
Study flowchart.

The sample size was calculated with free GRANMO software^1^ to detect a difference of 0.8 units in the Body Mass Index (BMI) between subjects who present or do not present any behavioral or substance addiction. A 1:3 ratio of people with addictions/non-addictions is estimated (1). Accepting an alpha risk of 0.05 and a beta risk of less than 0.2 in a bilateral contrast, 108 subjects are therefore needed in the first group and 360 in the second to detect a difference equal to or greater than 0.8 BMI units, assuming a common standard deviation of 2.16 BMI units (17). Therefore, the 500 subjects included will be sufficient to test the proposed hypotheses. The description of the subjects included in this study is shown in Figure 1. At the end of the study, each patient will be sent a detailed report with the results of the tests performed. In addition, a dissemination session will be organized for all patients included in the study.

### 2.3 Variables and measurement instruments

All measurements shall be carried out within a maximum period of 8 days. Researchers collecting data shall be trained in advance following a standardized protocol and quality assessment shall be carried out by an independent researcher.

Table 1 shows the questionnaires and tests performed on the subjects during the study.

**Table 1:** Summary of variables and form of measurement to be carried out in the study.

Sociodemographic variables	
Age, sex, marital status, education level and employment	
Anthropometric variables	
Body weight, height, Body mass index, waist circumference, blood pressure	
Body composition	Inbody 230^®^ multifrequency analyzer (Biospace)
Lifestyles	
Diet quality	Mediterranean Diet Adherence Screener (MEDAS)
Physical activity and sedentary time	International Physical Activity Questionnaire-Short Form (IPAQ-SF)
	Accelerometer (ActiGraph-GT3X)
	Marshall Sitting Questionnaire (MSQ)
Quality and quantity of sleep	Pittsburgh Quality Index (PSQI)
	Ford Insomnia Response to Stress Test
Behavioral addictions	
Gambling	Brief Questionnaire of Pathological Gambling.
	Lie/Bet Scale.
Internet use	Compulsive Internet Use Scale (CIUS)
Smartphone use	Smartphone Dependence and Addiction Scale (EDAS-18)
Video games use	Video Game Related Experiences Questionnaire (CERV)
Drugs use and abuse	
Smoking habits	4-item questionnaire adapted from the MONICA study (WHO)
	Fagerström test
Alcohol consumption	Questionnaire on alcohol consumption in the last 7 days
	Alcohol Use Disorders Identification Test (AUDIT)
Drug use	Alcohol, Smoking and Substance Involvement Screening Test (ASSIST V3.0)
	Drug Abuse Screening Test (DAST-20)
Vascular function and aging	
Carotid-femoral pulse wave velocity (cfPWV)	SphygmoCor device
Central augmentation index (CAIx)	SphygmoCor device
Cardio ankle vascular index (CAVI)	VaSera *VS*-2000^®^ device
Brachial-ankle pulse wave velocity (baPWV)	VaSera *VS*-2000^®^ device
Target organ injury	
Heart evaluation	ECG (General electric 5000)
Kidney evaluation	Serum creatinine, glomerular filtration rate and albumin-creatinine ratio
Cognitive alteration	
Cognitive function	Montreal Cognitive Assessment (MoCA) scale

#### 2.3.1 Sociodemographic variables and personal and family background

At the time of inclusion in the study, age, sex, marital status, educational level and current employment status will be collected. In addition, personal history of hypertension, hypercholesterolemia, thyroid problems or other diseases, drug use and whether they follow any special diet will be recorded. Direct family history of cardiovascular and cerebrovascular diseases will also be asked, as well as a history of drug use and addictions to gambling, mobile phones and/or social networks.

#### 2.3.2 Addiction assessment

##### 2.3.2.1 Behavioral addictions

The following addictions will be evaluated through questionnaires: *Betting with money* using the Cuestionario Breve de Juego Patológico (CBJP) (Short pathological gambling questionnaire) (50). This comprises four dichotomous items (yes/no) referring to gambling habits, feeling of guilt, inability to give up gambling, and use of household money for gambling. A score of 2 or more will be considered pathological gambling. The existence of problematic gambling will also be assessed with the Lie/Bet scale (51), consisting of two items, one referring to lies and the other to gambling; answering yes to both items will be considered problematic. *Internet use* will be assessed with the version of the Compulsive Internet Use Scale (CIUS) adapted to the Spanish population (52), validated for young people in this age range and with a cut-off score of 28. *Compulsive smartphone use* will be measured using the short version of the Escala de Dependencia y Adicción al Smartphone (EDAS-18) (Smartphone dependence and Addiction Scale) (53), validated in the adult population (54). *Videogame use* will be evaluated with the Cuestionario de Experiencias Relacionadas con Videojuegos (CERV) (Videogame-related Experiences Questionnaire) (55) which has two dimensions, one regarding negative consequences and the other about escapism. A score above 26 is considered to be potentially problematic for the user.

##### 2.3.2.2 Substance addiction

To assess addiction to drugs, we will use the following questionnaires: *Alcohol, Smoking and Substance Involvement Screening Test (ASSIST V3.0)*, consisting of eight items related to the use of nine substances, categorized into three risk levels: “low (0–3 points),” “moderate (4–26 points)” and “high (27 points and above)” (56, 57). The Spanish version of the *Drug Abuse Screening Test (DAST-20)* (58) consists of 20 questions with dichotomous answers (Yes/No), with 5 as a cut-off score. *Alcohol Use Disorders Identification Test (AUDIT)* consists of 10 questions allowing discrimination between risky use (8–15 points), harmful use (16–19 points) and alcohol dependence (20 points or above) (59). Using a structured questionnaire, the number of alcoholic drinks consumed in the previous week will be recorded, the grams/week consumed will be estimated and the patient will be classified as abstinent, low risk, intermediate risk or high risk, according to Spanish Ministry of Health criteria (60) on tobacco and alcohol consumption. Smoking will also be assessed with the standard four-item questionnaire adapted from the WHO MONICA study (61). The assessment of nicotine dependence uses the Fagerström test, comprising six questions that allows discrimination between mild dependence (under 4 points), moderate (4–6 points) or severe (7 points or over) (62).

#### 2.3.3 Anthropometric variables and blood pressure measurement

Height will be measured in cm with a calibrated measuring rod, with the patient inspiring, barefoot and with heels against the wall. Waist circumference shall be measured with a flexible tape measure, with the tape parallel to the floor above the iliac crests, at the end of expiration and with the patient standing upright and without clothing. The hip circumference shall be measured at the point of maximum circumference, passing through the greater trochanter of the two femurs. Body composition shall be measured by bioimpedance with the Inbody 230^®^ multifrequency analyzer (Biospace) according to the manufacturer’s instructions. Clinic blood pressure (BP) will be measured three times, using a validated Omron model M10-IT sphygmomanometer (Omron Healthcare, Kyoto, Japan). Measurements will be performed on the participant’s dominant arm in a sitting position after at least 5 min of rest with an appropriately sized cuff, determined by measuring the upper arm circumference and following the recommendations of the European Society of Hypertension (ESH) (39).

#### 2.3.4 Vascular function

Pulse wave analysis and carotid-femoral pulse wave velocity (VOPcf) with the Sphygmocor System^®^: with the patient in the supine position, the pulse wave in carotid and femoral arteries is analyzed, estimating the delay with respect to the ECG wave and calculating the VOPcf. Central and peripheral Augmentation index (AIx): with the patient seated and the arm resting on a rigid surface, pulse wave analysis is determined by a sensor in the radial artery, estimating the aortic pulse wave (63). CAVI and baPWV will be estimated with the *VaSera VS-2000* device *(Fukuda Denshi Co, Ltd, Tokio, Japón)* following the manufacturer’s instructions. Only CAVI measurements obtained for at least three consecutive heartbeats will be considered valid (64). The baPWV will be estimated using the following equation: baPWV = ((0.5934 × height(cm) + 14.4724))/tba, where tba is the time interval between the brachial and ankle waves (65). CAVI values will be divided into: normal (CAVI <8), normal-high (8 ≤ CAVI <9) and abnormal (CAVI ≥9) (64, 66).

#### 2.3.5 Vascular aging

Vascular aging will be assessed using cfPWV, baPWV or vascular age, estimated by the *VaSera VS-2000* device *(Fukuda Denshi Co, Ltd, Tokio, Japón)* as measures of stiffness. Firstly, subjects with vascular injury (carotid artery injury or peripheral artery disease) will be classified as EVA. In a second step, VAS is defined if the cfPWV or baPWV values are higher than the 90th percentile (p); EVN if they are between p10 and p90 and EVS if the values are below p10 (67, 68).

#### 2.3.6 Lifestyles

##### 2.3.6.1 Diet quality

The quality of the diet will be assessed with the validated 14-item Mediterranean Diet Adherence Screener questionnaire (MEDAS) (69) developed by the PREDIMED project: it sets out 14 items. Adherence to the Mediterranean diet is considered if the total score is ≥9 points.

##### 2.3.6.2 Physical activity and sedentary lifestyle

Physical activity will be assessed objectively for 7 days with the *ActiGraph-GT3X* accelerometer *(ActiGraph, Shalimar, FL)*, validated (70). The measurement includes daily step count, time and intensity of physical activity, and sedentary time in minutes per week for seven consecutive days. The intensity of physical activity (low, moderate or high) will be determined according to the cut-off points proposed by Freedson et al. (71). Intensity will also be measured subjectively with the *International Physical Activity Questionnaire-Short Form (IPAQ-SF)* (72). Sedentary behavior will be assessed with the *Marshall Sitting Questionnaire* (MSQ): it assesses daily sitting time in different activities: traveling or commuting, work/class, watching TV, using the computer at home and during leisure time (73).

#### 2.3.7 Sleep quality and quantity

*Pittsburgh Sleep Quality Index* (PSQI): assesses sleep quality and sleep disturbances over a one-month interval. Nineteen individual items generate seven “component” scores: subjective sleep quality, sleep latency, sleep duration, habitual sleep efficiency, sleep disturbances, sleep medication use and daytime dysfunction. The sum of the scores of these seven components results in an overall score. The cut-off point is 5, such that scores less than or equal to indicate good sleep quality, while higher values indicate poor sleep quality (74). The *Ford Insomnia Response to Stress Test* questionnaire to assess insomnia as a response to stress: it consists of nine questions about situations that can cause stress in the person and make it difficult to fall asleep (75).

#### 2.3.8 Target organ injury

##### 2.3.8.1 Kidney injury

The CKD-EPI formula: will be assessed with plasma creatinine, glomerular filtration rate estimated with the CKD-EPI formula (76), following the criteria of the ESC/ESH arterial hypertension management guidelines (39).

##### 2.3.8.2 Cardiac injury

Cardiac lesion will be assessed with a *General electric 5,000*^®^ digital ECG that automatically determines the voltage and wave duration, and estimates Cornell-Lyon and Cornell criteria to assess left ventricular hypertrophy (LVH) (39).

##### 2.3.8.3 Cognitive assessment

The *Montreal Cognitive Assessment (MoCA)*, a dementia screening tool validated in Spain (77) will be performed. Of the total possible score is 30 points; a score of 26 or more is considered normal.

##### 2.3.8.4 Analytical tests

Venous blood samples and urine samples will be taken between 08:00 and 09:00, with participants fasting, without smoking, drinking alcohol or caffeinated beverages, for the previous 12 h. On inclusion, an analysis will be performed to determine baseline blood glucose, urea, uric acid, creatinine, glomerular filtration rate estimated with the *CKD-EPI* equation, ionogram, thyroid function, lipid profile, blood count, liver profile, vitamin D and albumin-creatinine index. Samples will be taken in the APISAL Research Unit and sent by preferential internal delivery to the Clinical Analysis Service of the University Healthcare Complex of Salamanca in collaboration with the Biochemistry and Immunochemistry Section of the University Hospital of Salamanca. The samples will be coded and the laboratory techniques will be standardized.

### 2.4 Procedures

The investigators will phone potential candidates to participate in the study, explaining the purpose of the study. Those who agree to participate will be scheduled for an in-person visit, where they will be informed about the study and invited to sign the consent form. A fasting blood test will be performed at the same visit, as well as the rest of the test. Sociodemographic data, personal and family health history, smoking status, alcohol and drugs use, physical activity, sedentary time, Mediterranean diet, and cognitive evaluation will be collected. A clinical evaluation will be performed by recording blood pressure, height, weight, waist and hip circumference, body composition and vascular assessment tests. Finally, the accelerometer will be placed on the waist for 7 days and self-administered questionnaires will be given. The second visit will be arranged 8 days later for the return of both.

A report on the tests performed and the results of the analysis will be given to all participants. If any of the tests or questionnaires show altered results, patients will be referred to their family physician for follow-up, treatment or referral to the second level of care if necessary.

### 2.5 Statistical analysis

Data will be recorded using the REDCap platform (Research Electronic Data Capture) (78, 79) with a previously designed questionnaire. The normal distribution of the variables will be verified with the Kolmogorov–Smirnov test. Differences in means between variables in two categories will be carried out using a Student’s *t*-test or a Mann–Whitney U-test, as appropriate, while qualitative variables will be analyzed using an X^2^ test. To analyze the relationship between qualitative variables of more than two categories and quantitative variables, an analysis of variance will be used and in the *post hoc* analysis the LSD will be used. The Kruskal-Wallis test will be used in cases where the variables are not normally distributed. Analysis of covariance (ANCOVA) will be performed to adjust for variables that may affect the results as confounding factors. The relationship of the quantitative variables with each other will be tested using the Pearson or Spearman correlation, as appropriate. To analyze the relationship of the different addictions with obesity, physical activity, sedentary lifestyle and arterial stiffness, a multiple regression analysis will be performed. Logistic regression will be used to assess the association between having or not having addictions with obesity, physical activity, sedentary lifestyle and vascular aging, adjusted for possible confounding variables. Data will be analyzed using the statistical package SPSS Windows version 26.0. (IBM, Armonk, New York: IBM Corp.). A *p* < 0.05 will be considered statistically significant and in the case of multiple comparisons the Bonferroni correction will be done. The statisticians/researchers performing the different analyses will be blinded to the patient’s clinical data. All variables will be analyzed disaggregated by sex, and where appropriate, differences will be analyzed from a gender perspective, as the influence of gender on numerous pathologies, particularly cardiovascular and cerebrovascular diseases, is well known.

## 3 Discussion

Behavioral and substance addictions have become progressively more prevalent (3), while in parallel, accelerated vascular aging has also occurred. Thus, finding relationships between behavioral and substance addictions with vascular stiffness and aging, as well as with sleep, physical activity, and cognitive impairment may shed light on this field in order to design future interventions for young adults.

Behavioral addictions, such as gambling, internet and cell phone use, and video games, are rising among young adults (1). They are associated with unhealthy lifestyles and increase health problems such as eye strain, tiredness, headaches and obesity, disrupt sleep quantity and quality, and cause withdrawal symptoms (anger and irritability) and even substance abuse due to compulsive disorder, leading to mental health problems (4, 5). Thus, the increased use of smartphones is closely linked to low self-esteem and the need for self-control, which is why adolescents seek support on social networks as a stress coping mechanism to alleviate depression and anxiety (80). The time spent on screens has risen progressively, as has the prevalence of gaming disorder, in parallel with the increase in substance use (81, 82). These facts mean more sedentary time and, therefore, less physical activity (83), greater obesity (19, 84), worse sleep quality and quantity (85), all of which are risk factors for accelerated vascular aging.

Meanwhile, substance addictions are linked to lifestyle in various ways. Thus, lifestyles can influence the development of a substance addiction, and in turn, addiction can affect and modify lifestyles. One study has shown how participants with unhealthy lifestyles were associated with substance use (86). Moreover, some substances, such as alcohol, are a risk factor for obesity (87). Substance addictions, especially those that affect the cardiovascular system, have a significant impact on vascular aging. Using cocaine or amphetamines increases blood pressure and in turn can accelerate vascular aging (88). Smoking also accelerates vascular aging (68). Excessive and chronic alcohol use contributes to premature aging of blood vessels (89) and many substances, including some illicit substances, can generate oxidative stress and an inflammatory response in the cardiovascular system, increasing vascular aging (90). In summary, substance addictions can increase the risk of developing cardiovascular diseases such as hypertension, coronary heart disease and stroke, diseases which are related to vascular aging and can accelerate the deterioration of blood vessels. Finally, it must not be forgotten that polydrug use increases risks, heightening the effects of some substances on others, reinforcing addiction, interfering with diagnosis, making treatment difficult and increasing mental illnesses (10).

In conclusion, all of the above makes it necessary to carry out studies which relate the presence of addictions, both behavioral and substance, to the increase in obesity and sedentary lifestyle, reduced physical activity, sleep patterns, and accelerated vascular aging. The results of this project will allow us to better understand the situation regarding behavioral and substance addictions in young adults in the urban health area of Salamanca. In addition, it will analyze how addictions are linked to lifestyles, arterial stiffness and vascular aging, being to the best of our knowledge the first study to analyze the relationship between addictions and vascular aging in a sample of young adults, as well as the effects they have on obesity and lifestyles. The evidence generated will thus allow the development of preventive/therapeutic strategies to modify lifestyle from an early age and thus contribute to healthy vascular aging. All of this will allow us to learn which participant profiles are at highest risk of accelerated vascular aging so that this can be transferred to clinical practice. This can contribute to the development of personalized medicine, adapting preventive/therapeutic interventions to each user, thus generating an individual approach, based on the specific characteristics of each person.

The main limitations of the study are: firstly, since it is a random sample collected in the urban area of Salamanca, the data cannot be extrapolated to the rural population. Secondly, causality cannot be assumed as it is a cross-sectional study, but associations can be analyzed and hypotheses generated for future prospective etiological studies with a larger sample of subjects. On the other hand, this project also has strengths in that it is the first that attempts to link addictions to lifestyle and accelerated vascular aging in a young adult population, and its results will allow evidence to be generated for the design of preventive interventions in this age range.

## 4 Brief summary

Behavioral and substance addictions affect a significant percentage of the population between the ages of 18–34. Likewise, obesity and unhealthy lifestyles such as decreasing physical activity and increasing sedentary time, mostly spent in front of screens, plus a rise in the consumption of processed foods, are increasing in this population group. These factors are related to disturbed sleep quantity and quality, increased obesity, and may be related to arterial stiffness and early vascular aging.

For the above reasons, we propose this study with the main objective of analyzing the association of addictions with obesity and the distribution of body fat, physical activity, sedentary time, arterial stiffness and vascular aging, sleep quality and cognitive function, and gender differences in young adults aged between 18 and 34. Studying all these variables assessed with validated questionnaires or validated objective examinations or tests will allow us to identify people with a higher risk of presenting behavioral addictions or substance addictions. A deeper knowledge of all these health problems and the relationship between them can facilitate the development of personalized medicine, adapting preventive/therapeutic interventions with an individual approach and early and appropriate clinical support.

## 5 Ethics and dissemination

### 5.1 Ethical approval and consent for participation

The study was approved by the Committee of ethics of research with medicines of the health area of Salamanca on 7/10/2021 (CEIm reference code Ref. PI 2021 088671048), and 24/07/2023 (CEIm reference code Ref. PI 2023 071332). Before the start of the study, all participants will sign the informed consent (Supplementary material 2). During the development of the study, the standards of the Declaration of Helsinki (91) and the WHO guidelines for observational studies will be followed. Subjects will be informed of the objectives of the project and the risks and benefits of the explorations to be carried out. The study does not contemplate any intervention entailing a risk greater than the minimum involved in carrying out the different tests. All information generated in this study will be stored, encrypted and used exclusively for the purposes specified here. Both the samples and the data collected will be associated with a code and stored under appropriate security conditions, and participants are guaranteed that they cannot be identified through means considered reasonable by persons other than those authorized. The confidentiality of the participants included will be guaranteed at all times in accordance with the provisions of Organic Law 3/2018, December 5, regarding the Protection of Personal Data and guarantee of digital rights and Regulation (EU) 2016/679 of the European Parliament and of the Data Protection Council of April 27, 2016 (GDPR).

The participants will not receive any compensation for the visit completion. They will receive a report of tests performed on the state of their health status.

### 5.2 Dissemination plan

The data will be available to the members of the research group, who will be mainly responsible for dissemination. In addition, the variables used in each manuscript will be available to the entire scientific community through the Gredos scientific repository of Salamanca University. The results of the study will be published in peer-reviewed open access scientific journals, to be complemented by the presentation of the study results at national and international scientific conferences. Likewise, suitable dissemination will be carried out through social networks, information days for citizens, in other media and directly to those participating in the study. Current and future knowledge of the relationship between addictions and lifestyle, and how this relationship affects accelerated vascular aging in young adults will be transferred to clinical practice.

## Data availability statement

The original contributions presented in the study are included in the article/Supplementary material, further inquiries can be directed to the corresponding author.

## Ethics statement

The studies involving humans were approved by The Ethics Committee for Research with Medicines of the Salamanca Health Area. The studies were conducted in accordance with the local legislation and institutional requirements. The participants provided their written informed consent to participate in this study. Written informed consent was obtained from the individual(s) for the publication of any potentially identifiable images or data included in this article.

## Author contributions

SV-G: Writing – original draft. CL-S: Writing – original draft. OT-M: Writing – review & editing. AV: Writing – review & editing. SG-S: Writing – review & editing. SC: Writing – review & editing. MG-S: Writing – review & editing. ER-S: Writing – review & editing. LG-O: Writing – review & editing. LG-S: Supervision, Writing – review & editing. MG-M: Supervision, Writing – review & editing. EVA-Adic Investigators Group: Investigation, Writing – review & editing.

## EVA-Adic Investigators Group

The members of the EVA-Adic Group are: Manuel A. Gómez-Marcos, Luis García-Ortiz, Emiliano Rodríguez-Sánchez, Cristina Lugones-Sánchez, Olaya Tamayo-Morales, Susana González-Sánchez, Leticia Gómez-Sánchez, Sara M. Vicente-Gabriel, Alberto Vicente-Prieto, Sandra Conde-Martín, Marta Gómez-Sánchez, Elena Navarro-Matias, Carmen Patino-Alonso, José A. Maderuelo-Fernández, Angela de Cabo-Laso, Benigna Sanchez-Salgado, and Laura Fernandez-Matas.

## Figures and Tables

**Table:** 

ANOVA	Analysis of variance
APISAL	Primary Care Research Unit of Salamanca
AS	Arterial stiffness
ASSIST	The Alcohol, Smoking and Substance Involvement Screening Test
AUDIT	Alcohol Use Disorders Identification Test
baPWV	Brachial-ankle pulse wave velocity
BMI	Body Mass Index
BP	Blood Pressure
CA	Cardiovascular aging
CAIx	Central augmentation index
CAUSA	Complejo Asistencial Universitario de Salamanca
CAVI	Cardio-ankle vasculat index
CBJP	El Cuestionario Breve de Juego Patológico
CEIm	Comité de Ética Investigación con Medicamentos
CERV	The Questionnaire of Experiences Associated with Video games
cfPWV	Carotid-femoral pulse wave velocity
CIUS	Compulsive Internet Use Scale
CKD-EPI	Chronic Kidney Disease Epidemiology Collaboration
DAST	Drug Abuse Screening Test
DP	Diastolic pressure
EDAS	Escala de dependencia y adicción al smartphone
EDADES	Encuestas sobre Alcohol y Otras Drogas en España
EKG	Electrocardiogram
ESH	European Society of Hypertension
EU	European union
EVA	Early vascular aging
GRS	Gerencia Regional de Salud
HVA	Healthy vascular aging
ISCIII	Instituto de Salud Carlos III
IBSAL	Instituto de Investigación Biomédica
IPAQ	International physical activity questionnaire
LVH	Left ventricular hypertrophy
MEDAS	Mediterranean Diet Adherence Scale
MET	Equivalentes metabólicos de tarea
MoCA	Montreal Cognitive Assessment
MRR	Facility for Recovery and Resilience
MSQ	Marshall Sitting Questionnaire
NVA	Normal vascular aging
OMS	Organización Mundial de la Salud
PDV	Product of Duration by Voltage
PREDIMED	Prevención con Dieta Mediterránea
PSQI	Pittsburgh’s sleep quality index
RGPD	Reglamento de Protección de Datos
RICAPPS	Network of Research in Chronicity, Primary Care and Health Prevention and Promotion
SP	Systolic pressure
SPIRIT	Standard Protocol Items Recommendations for Interventional Trials
TBA	Time interval between arm and ankle waves
TSI	Tarjeta Sanitaria Individual

## Footnotes

^1^http://www.imim.cat/ofertadeserveis/software-public/granmo/
