# Supplementary material for: Relationship between addictions and obesity, physical activity and vascular aging in young adults (EVA-Adic study): a research protocol of a cross-sectional study
Source: Front Public Health. 2024 Jan 26;12:1322437. doi: 10.3389/fpubh.2024.1322437 (PMC10853417; doi:10.3389/fpubh.2024.1322437)
Supplement: Supplementary file 1 [file Data_Sheet_1.PDF]

ANEXO 1: SPIRIT CHECKLIST.

Descripción de la información administrativa.

1. Title. Descriptive title identifying the study design, population, interventions, and, if applicable, trial acronym	☒
2. Trial registration 2a Trial identifier and registry name. If not yet registered, name of intended registry<br>2b All items from the World Health Organization Trial Registration Data Set	☒
3. Protocol versión. Date and version identifier	☒
4. Funding. Sources and types of financial, material, and other support	☒
5. Roles and responsibilities. 5ª Names, affiliations, and roles of protocol contributors.<br>5b Name and contact information for the trial sponsor<br>5c Role of study sponsor and funders, if any, in study design; collection, management, analysis, and interpretation of data; writing of the report; and the decision to submit the report for publication, including whether they will have ultimate authority over any of these activities.<br>5d Composition, roles and responsibilities of the coordinating centre, steering committee, endpoint adjudication committee, data management team, and other individuals or groups overseeing the trial, if applicable.	☒

Introduction

6. Background and rationale. 6a Description of research question and justification for undertaking the trial, including summary of relevant studies (published and unpublished) examining benefits and harms for each intervention.<br>6b Explanation for choice of comparators.	☒
7. Objectives. Specific objectives or hypotheses	☒

8. Trial design. Description of trial design including type of trial (eg, parallel group, crossover, factorial, single group), allocation ratio, and framework (eg, superiority, equivalence, noninferiority, exploratory).	☒

Methods: Participants, interventions, and outcomes

9. Study setting. Description. Of study settings (eg, community clinic, academic hospital) and list of countries where data will be collected. Reference to where list of study sites can be obtained.	☒
10. Eligibility criteria. Inclusion and exclusion criteria for participants. If applicable, eligibility criteria for study centres and individuals who will perform the interventions (eg, surgeons, psychotherapists).	☒
11. Interventions. 11a Interventions of each group with sufficient detail to allow replication, including how and when they will be administered.<br>11b Criteria for discontinuing or modifying allocated interventions for a given trial participant (eg, drug dose change in response to harms, participant request, or improving/worsening disease).<br>11c Strategies to improve adherence to intervention protocols, and any procedures for monitoring adherence (eg, drug tablet return, laboratory tests).<br>11d Relevant concomitant care and interventions that are permitted or prohibited during the trial.	☒
12. Outcomes. Primary, secondary, and other outcomes, including the specific measurement variable, analysis metric, method of aggregation, and time point for each outcome. Explanation of the clinical relevance of chosen efficacy and harm outcomes is strongly recommended.	☒
13. Participant timeline. Time schedule of enrolment, interventions (including any run-ins and washouts), assessments, and visits for participants. A schematic diagram is highly recommended.	☒
14. Sample size. Estimated number of participants needed to achieve study objectives and how it was determined, including clinical and statistical assumptions supporting any sample size calculations.	☒

15. Recruitment. Strategies for achieving adequate participant enrolment to reach target sample size.	☒

Methods: Assignment of interventions (for controlled trials)

16. Allocation. 16a Sequence generation. Method of generating the allocation sequence, and list of any factors for stratification. To reduce predictability of a random sequence, details of any planned restriction should be provided in a separate document that is unavable to those who enrol participants or assign interventions.<br>16b Allocation concealment mechanism. Mechanism of implementing the allocation sequence (eg, central telephone; sequentially numbered, opaque, sealed envelopes), describing any steps to conceal the sequence until interventions are assigned.<br>16c Implementation. Who will generate the allocation sequence, who will enrol participants, and who will assign participants to interventions.	☒
17. Blinding (masking). 17a Who will be blinded after assignment to interventions, and how.<br>17b If blinded, circumstances under which unblinding is permissible, and procedure for revealing a participant´s allocated intervention during the trial.	☒

Methods: Data collection, management, and analysis

18. Data collection methods. 18a Plans for assessment and collection of outcome, baseline, and other trial data, including any related processes to promote data quality and a description of study instruments along with their reliability and validity, if known. Reference to where data collection forms can be found, if not in the protocol.<br>18b Plans to promote participant retention and complete follow-up, including list of any outcome data to be collected for participants who discontinue or deviate from intervention protocols.	☒
19. Data management. Plans for data entry, coding, security, and storage, including any related processes to promote data quality (eg, double data entry; range checks for data values). Reference to where details of data management procedures can be found, if not in the protocol.	☒

20. Statistical methods. 20a Statistical methods for analysing primary and secondary outcomes. Reference to where other details of the statistical analysis plan can be found, if not in the protocol.<br>20b Methods for any additional analyses (eg, subgroup and adjusted analyses).<br>20c Definition of analysis population relating to protocol non-adherence, and any statistical methods to handle missing data.	☒

Methods: Monitoring

21. Data monitoring. 21a. Composition of data monitoring committee (DMC); summary of its role and reporting structure; statement of whether it is independent from the sponsor and competing interests; and reference to where further details about its charter can be found, if not in the protocol. Alternatively, an explanation of why a DMC is not needed.<br>21b Description of any interim analyses and stopping guidelines, including who will have access to these interim results and make the final decision to terminate the trial.	☒
22. Hams. Plans for collecting, assessing, reporting, and managing solicited and spontaneously reported adverse events and other unintended effects of trial interventions or trial conduct.	☒
23. Auditing. Frequency and procedures for auditing trial conduct, if any, and whether the process will be independent from investigators and the sponsor.	☒

Ethics and dissemination

24. Research ethics approval. Plans for seeking research ethics committee/institutional review board (REC/IRB) approval.	☒
25. Protocol amendments. Plans for communicating important protocol modifications (eg, changes to eligibility criteria, outcomes, analyses) to relevant parties (eg, investigators, REC/IRBs, trial participants, trial registries, journals, regulators).	☒

26. Consent or assent. 26a Who will obtain informed consent or assent from potential trial participants or authorised surrogates, and how (see Item 32).<br>26b Additional consent provisions for collection and use of participant data and biological specimens in ancillary studies, if applicable.	☒
27. Confidentiality. How personal information about potential and enrolled participants will be collected, shared, and maintained in order to protect confidentiality before, during, and after the trial.	☒
28. Declaration of interests. Financial and other competing interests for principal investigators for the overall trial and each study site.	☒
29. Access to data. Statement of who will have access to the final trial dataset, and disclosure of contractual agreements that limit such access for investigators.	☒
30. Ancillary and post-trial care. Provisions, if any, for ancillary and post-trial care, and for compensation to those who suffer harm from trial participation.	☒
31. Dissemination policy. 31a Plans for investigators and sponsor to communicate trial results to participants, healthcare professionals, the public, and other relevant groups (eg, via publication, reporting in results databases, or other data sharing arrangements), including any publication restrictions.<br>31b Authorship eligibility guidelines and any intended use of professional writers.<br>31c Plans, if any, for granting public access to the full protocol, participant-level dataset, and statistical code.	☒

Appendices

32. Informed consent materials. Model consent form and other related documentation given to participants and authorised surrogates.	☒
33. Biological specimens. Plans for collection, laboratory evaluation, and storage of biological specimens for genetic or molecular analysis in the current trial and for future use in ancillary studies, if applicable.	☒
